# Persistently high prevalence of HPV16 and rising prevalence of non-16/18 HR-HPV genotypes in cervical precancer and cancer in Latvia in 2016-2024 shape the severity of cervical disease

**DOI:** 10.3389/fonc.2025.1676334

**Published:** 2026-02-04

**Authors:** Liba Sokolovska, Karina Biserova, Arta Spridzane, Daira Krisane, Alesja Dudorova, Svetlana Gebrila, Ilvija Krasovska, Dmitry Perminov, Beatrise Orlova, Marta Petrovska, Juris Jansons, Androniks Mitildzans, Jurijs Nazarovs, Maria Isaguliants

**Affiliations:** 1 Institute of Microbiology and Virology, Riga Stradins University, Riga, Latvia; 2 Pathology Institute, Pauls Stradins Clinical University Hospital, Riga, Latvia; 3 Oncology Centre of Latvia, Riga East Clinical University Hospital, Riga, Latvia; 4 Pathology Centre, Riga East Clinical University Hospital, Riga, Latvia; 5 E. Gulbis Laboratory Ltd, Riga, Latvia; 6 Centrala Laboratorija Ltd, Riga, Latvia; 7 Latvian Biomedical Research and Study Center, Riga, Latvia; 8 Department of Pathology, Riga Stradins University, Riga, Latvia; 9 Department of Microbiology, Tumor and Cell Biology, Karolinska Institutet, Stockholm, Sweden

**Keywords:** cervical cancer, cervical dysplasia, HR-HPV, infection, genotyping

## Abstract

**Background:**

Cervical cancer incidence and mortality in Latvia is one of the highest in Europe, but data on HR-HPV prevalence in cervical disease are lacking. We aimed to investigate HR-HPV prevalence in cervical squamous cell carcinoma (CSCC) and cervical dysplasia (CD), association with disease severity, and prevalence changes over time.

**Materials & Methods:**

Cervical tissue samples from 145 patients were retrieved and used for HR-HPV genotyping using two commercially available PCR kits.

**Results:**

Only six CD samples were HR-HPV negative (6/66, 9.1%), while all CSCC were positive. Over 50% samples (75/139) harbored one, 33.8% two, 10.1% three, and 2.2% four HR-HPV genotypes. CSCC was more likely to harbor multiple HR-HPVs (p=0.0280). HPV16 remained most prevalent in CSCC and CD and was followed by HPV33 (32/139, 23.0%), HPV39 (13/139, 9.4%), and HPV18 (11/139, 7.9%). CSCC samples were more likely to have high HR-HPV loads (p=0.025). Disease severity expressed as CINI to CSCC grade 3, correlated with HR-HPVs detected (p=0.015, r=0.202) and HPV16 and HPV39 loads (p<0.001, r=0.354; p<0.001, r=0.307). prevalence decreased, insignificantly across the analyzed period, while HPV18 decreased significantly (2016-18: 17.4% vs. 2022-24: 2.2%; p=0.032). HPV66, 45, 39, 31, and 33 (2016-18: 13%; 2022-24:31.1%; p=0.045) increased.

**Discussion:**

HPV16 remained the most prevalent HR-HPV, while HPV18 decreased. Other HR-HPV genotypes (HPV 66/45/39/31/33) demonstrated an increase in prevalence. Cervical disease severity was linked to specific HR-HPV loads and the number infecting HR-HPVs. These findings highlight the need for extended HR-HPV genotyping with determination of viral load, and request more epidemiological studies analyzing historical and current samples.

## 1 Introduction

For years, the groundbreaking discovery of the high-risk human papillomavirus (HR-HPV) association with cervical cancer development has been utilized in national screening and immunization campaigns and has contributed to a considerable reduction in cervical cancer mortality (1). Despite this, in many middle-income countries, cervical cancer incidence and associated mortality remain high (2). Latvia has one of the highest cervical cancer incidence and mortality rates in Europe, which is unfortunately accompanied by the relatively low screening rates and low HPV vaccine acceptance and uptake (3, 4).

Mass HPV immunization was shown to contribute to changes in HR-HPV genotype prevalence, where vaccine-targeted genotypes decrease in prevalence while non-vaccine genotypes increase (5, 6). Several studies have demonstrated the decline of HPV16/18 prevalence with the increase of prevalence of other HR-HPV genotypes, specifically those not included into HPV vaccine (7–9). While a study using a simulation model suggested that epidemiological studies conducted may not be powerful enough to detect true changes in the prevalence of non-vaccine genotypes, and that true changes could only be observed more than 10 years after the introduction of vaccination (10), the changes, so called “genotype shift”, were reported worldwide within few years after the on-start of mass vaccination (11, 12) This signifies the need for dynamic monitoring of the changes in HR-HPV genotype prevalence, data of extreme importance to guide national and worldwide HPV vaccination efforts.

In Latvia, HPV vaccination was introduced in 2010 using the bivalent Cervarix vaccine, targeting HPV16 and 18. In 2020, Latvia introduced the nonavalent Gardasil 9 vaccine, targeting HPV6, 11, 16, 18, 31, 33, 45, 52, and 58 (13). HR-HPV prevalence studies conducted in the Latvian population so far have primarily been based on the cervical smear samples, in women undergoing routine screening. No studies have monitored the changes in HR-HPV genotypes over time, specifically in women with cervical disease. Here, we aimed to investigate HR-HPV genotype prevalence and viral load in cervical tissues of CD and CSCC cases, associate them with disease severity, and assess how the genotype prevalence has changed over time.

## 2 Materials and methods

### 2.1 Patients & samples

Study was performed based on the ethical permit issued by the Riga Stradins University Ethical Committee (N2-PĒK-4/415/2022). The study assessed a panel of cervical tissue samples in the form of the formalin-fixed paraffin-embedded (FFPE) blocks, collected from the archive of the Pauls Stradins Clinical University Hospital between 2016 and 2024. Initial tissue samples were remitted to the hospital by the gynecologists/surgeons to confirm the suspected diagnosis of cervical dysplasia (CD) or cervical cancer (CC). Registry data on the samples included age of the patient, suspected diagnosis by the referring medical doctor, and diagnosis established on the basis of analysis made at the hospital.

Criteria to include samples into the retrospective study were: (i) preliminary diagnosis of cervical dysplasia (CD), including mild, moderate or severe dysplasia, or cervical squamous cell carcinoma (CSCC); (ii) confirmation of the diagnosis by certified pathologist at the hospital, assigning the case with ICD codes N87.0, N87.1, N87.2, C53, and C53.9, according to ICD-10-CM in-forced 2016 (14); (iii) size of FFPE block allowing at least 25 consecutive sections of 4 mm thickness with 50% of the block remaining thereafter (for eventual retrospective analysis upon request by the patient and/or her physician). The study recruited all archived samples that fulfilled the above criteria. CC cases not involving squamous epithelium, such as adenocarcinomas, were excluded as a heterogeneous group of tumors with varying morphologies, etiologies, and molecular drivers (15).

Written informed consent was not obtained from the individuals for the publication of any data included in this article because the study was retrospective, non-interventional, did not interfere with routine diagnosis and treatment, did not affect any medical rights of the patients, and did not add any additional risks to the patients.

### 2.2 HR-HPV genotyping in cervical tissues

The FFPE blocks were sectioned into 5 to 11 4-micron sections, which were processed to extract DNA as described by us earlier (16). DNA extraction was performed using the QIAamp DNA FFPE Advanced UNG kit (Qiagen) according to the manufacturer’s recommendations. DNA purity was assessed spectrophotometrically by measuring A260/A280 ratio.

Isolated DNA was used to detect 14 HR-HPV genotypes (HPV16, 18, 31, 33, 35, 39, 45, 51, 52, 56, 58, 59, 66, and 68) using either the semiquantitative Anyplex™ II HPV HR recording viral load as low(+), medium (++), high (+++) (85/145 samples) or the quantitative Allplex™ HPV HR Detection kit recording viral load as Ct values (60/145 samples) (both Seegene, Seoul, Republic of Korea). Both kits included an internal control (IC) based on the human beta globin gene. Successful amplification of the IC confirmed good DNA quality. Results from samples where IC failed to amplify were discarded.

To translate the Ct values provided by the Allplex kit into the context of semi-quantitative HR HPV amounts generated by Anyplex, we have built calibration curves using plasmid pLJM1_pGK_E6E7 DNA containing full-length copies of HPV16 E6 and E7 genes, recloned from a plasmid carrying the genome of HPV16 reference strain NC_001526.4 (pHPV16; www.atcc.org/products/45113d) (16). Plasmid pLJM1_pGK_E6E7 was diluted from 10^6^ to 1 copy in 10-fold serial steps. The number of E6E7 DNA copies in the sample was calculated as: DNA amount (ng) x 6.022x1023/length of DNA (bp) x 1x109 ng/ml x 650 Daltons (17). In-house PCR for HPV16 E6 DNA was performed as described earlier (16). The calibration curves were built attributing the cycle threshold (Ct) in in-house PCR, in Allplex, and semi-quantitative virus load in AnyPlex, to the number of copies of plasmid carrying E6E7 DNA. According to this calibration, low values (+) corresponded to Ct values >40 cycles, intermediate (++) to Ct values in the range of 30–38 cycles, and high (+++) to Ct values below 30 cycles. This calibration data fell in line with the data published by Oštrbenk Valenčak A et al. in 2018 and 2024, where low (+) was attributed to positive after 39 PCR cycles, intermediate (++), to positive within 31 to 39 cycles, and high (+++), to positive before 31 cycles for all HR HPVs (18, 19).

### 2.3 Data statistical analysis

Statistical data analysis and graphical representation were performed using GraphPad Prism (10.0.0 for MacOS, GraphPad Software, Boston, Massachusetts USA) for Fisher’s exact test and non-parametric Spearman correlation, and JASP (Version 0.19.3 for MacOS, JASP Team (2024)) for non-parametric Spearman correlation.

## 3 Results

### 3.1 Study population

FFPE cervical tissue blocks corresponding to CSCC (79/145, 54.5%) and CD cases (66/145, 45.5%), sampled between 2016 and 2024, were retrieved from the hospital repository. Patient data (diagnosis, disease grading, sampling year) are summarized in **Table 1**.

**Table 1:** Panel of cervical tissue samples included in the study of HR HPV prevalence in tissues of Latvian women with cervical disease collected between January 2016 and May 2024.

	Number of patients										Age (IQR)	
	Total	2016	2017	2018	2019	2020	2021	2022	2023	2024		
Total	145	14	14	18	17	16	21	15	26	4	46	(36-62)
Cervical squamous cell carcinoma	79	5	11	11	10	9	7	11	12	3	59	(49.5-70)
Grade 1	7	1	1	0	0	0	1	2	2	0	46	(41.5-76.5)
Grade 2	46	3	7	4	3	5	6	8	10	0	60	(51.25-70)
Grade 3	26	1	3	7	7	4	0	1	0	3	60.5	(52-67.25)
Cervical dysplasia	66	8	3	7	7	7	14	4	14	1	35.5	(30-41.75)
CIN I	18	0	0	1	0	3	5	2	7	0	39	(32-45.25)
CIN II	20	3	0	0	2	1	5	2	5	1	37	(30-39.25)
CIN III	28	5	3	6	5	3	4	0	2	0	33	(29-43.25)

Patients are divided into two diagnostic categories: cervical squamous cell carcinoma (CSCC) of grades G1 to G3, or cervical intraepithelial neoplasia (CIN) of grades I to III. Demographic data included median age of the patients with interquartile range (IQR).

The median age of patients at the time of sampling was 46 years (CD: 35.5 years; CSCC: 59 years). While CD patients were distributed relatively equally between the stages of the disease (CIN I: 18/66, 27.3%; CIN II: 20/66, 30.3%; CIN III: 28/66, 42.4%), the majority of CSCC patients had cancer grade 2 (58.2%, 46/79), while Grade 1 constituted 8.9% (7/79) and Grade 3, 32.9% (26/79) cases (**Table 1**). Information regarding previous treatment, screening or HPV vaccination status of the patients was not available. Of the 145 participants, only two were in the age category eligible to receive the HPV vaccine when the national vaccination campaign was initiated; thus, the majority (98.6%) was considered as not vaccinated.

### 3.2 Comparison of HR-HPV genotyping results - Anyplex™ vs Allplex™

During the first year of the study, HR-HPV genotyping was done using the AnyPlex kit, and starting from 2024, by AllPlex, as quantitative assessment of HR HPV load is desirable for prognosis of disease outcome. A direct comparison of the Allplex and Anyplex assays done earlier found no significant overall difference in sensitivity of HPV detection, with studies showing high agreement and no major differences in the genotype prevalence or overall detection rates (20, 21) promising smooth transition from semi-quantitative to quantitative PCR. A weak point was the necessity to combine the results obtained using two tests in the throughout analysis.

The genotyping results obtained by the Allplex™ kit were interpreted using the Ct cut-off value provided in the kit manual. With this, samples with a Ct value higher than 43 were interpreted as HPV negative. Initial translation of Allplex™ results into Anyplex™ ranges was done using the calibration curve built using HPV16 DNA (16). In this initial translation, Allplex™ results translated into AnyPlex™ and AnyPlex™ yielded similar results in 50% cases (9/18; **Table 2**). In three cases, Allplex™ detected genotypes that Anyplex™ had missed, and in one case, Anyplex™ detected HPV33 and HPV18, and Allplex™, HPV33 and HPV16 (**Table 2**, samples No. 5, 6, 8, and sample 7, respectively). Of note, all genotype discrepancies were in samples low viral loads. Resolving the conflict, a recently published article described new genotype-specific cut-off values recommended by AllPlex manufacturer: for HPV16 and 18, Ct >40; for HPV31, 33, 45, 52, and 58, Ct>37; for HPV35, 39, 51, 56, 59, 66, and 68, Ct >35 (19). These cut-offs reduced genotyping discrepancies to 1 out of 18 samples (5,5%), namely sample No. 7, for which AnyPlex reported HPV18 and HPV 33, and AllPlex, HPV16 and HPV33 (**Table 2**). Discrepancies still remained with respect to the viral loads, AnyPlex™ detecting overall lower viral loads than AllPlex (**Table 2**). Such “technical” reduction of viral load by AnyPlex™ was potentially reducing the significance of associations between viral load and disease severity, but had to be dealt with, in order to perform throughout data analysis.

**Table 2:** Comparison of HR-HPV genotyping results obtained using the Anyplex™ II HPV HR and the Allplex™ HPV HR Detection kits.

Sample	Anyplex™ II HPV HR detection	Allplex™ HPV HR detection		Verification of HPV16 test results by calibration curve - Low (+) Ct >39, Medium (++) Ct 30-38, high (+++)Ct <30	Reconstructed AnyPlex genotyping results based the recommended cut-offs – HPV16/18 Ct<40; HPV31/33/45/52/58 Ct<37; HPV35/39/51/56/59/66/68 Ct <35 (16, 18, 19)
		HR-HPV genotype	Ct value		
1	66+++	66	17,4		66
	51++	51	26,69		51
	16+	16	35,79	16++ ⇧	16
2	16+	16	29,57	16++ ⇧	16
3	45++	45	24,04		45
	16+	16	35,27	16++ ⇧	16
4	16+	16	33,83	16++ ⇧	16
5	16+	16	34,96	16++ ⇧	16
	18+	18	30,54		18
		33 ○	38,16		neg
6	52+	52	23,32		52
	35+	35	36,41		35
		33 ○	39,94		neg
7	33+	33	37,22		33
		16○	39,68	16+ ○	16
	18+ ○				neg
8	18++	18	31,22		18
		16 ○	40,49	16+ ○	neg
		33 ○	41,17		neg
9	16+	16	34,73	16++ ⇧	16
	18+	18	27,04		18

Allplex™ Ct values were translated to 3 levels used by the Anyplex kit based on the previously published data (16, 18, 19) and the calibration curve for HPV16 (see Materials and Methods section). Pluses indicate the relative amount of HPV (+++ - High, ++ - Medium, + - Low). ⇧ - reported higher HPV amount; ○ - reported different HPV genotype.

### 3.3 HR-HPVs in cervical cancer and dysplasia

#### 3.3.1 Multiple HR-HPV genotypes in cervical tissues

In the combined analysis by AnyPlex™ and AllPlex™ kits, out of the 145 cervical tissues, only six were found to be HR-HPV-negative (6/145, 4.1%). All CSCC samples, regardless of cancer grade, were positive for at least one HR-HPV genotype. All six HR-HPV negative samples originated from CD tissues (CIN I: 4/6; CIN III: 2/6).

**Figure 1:**
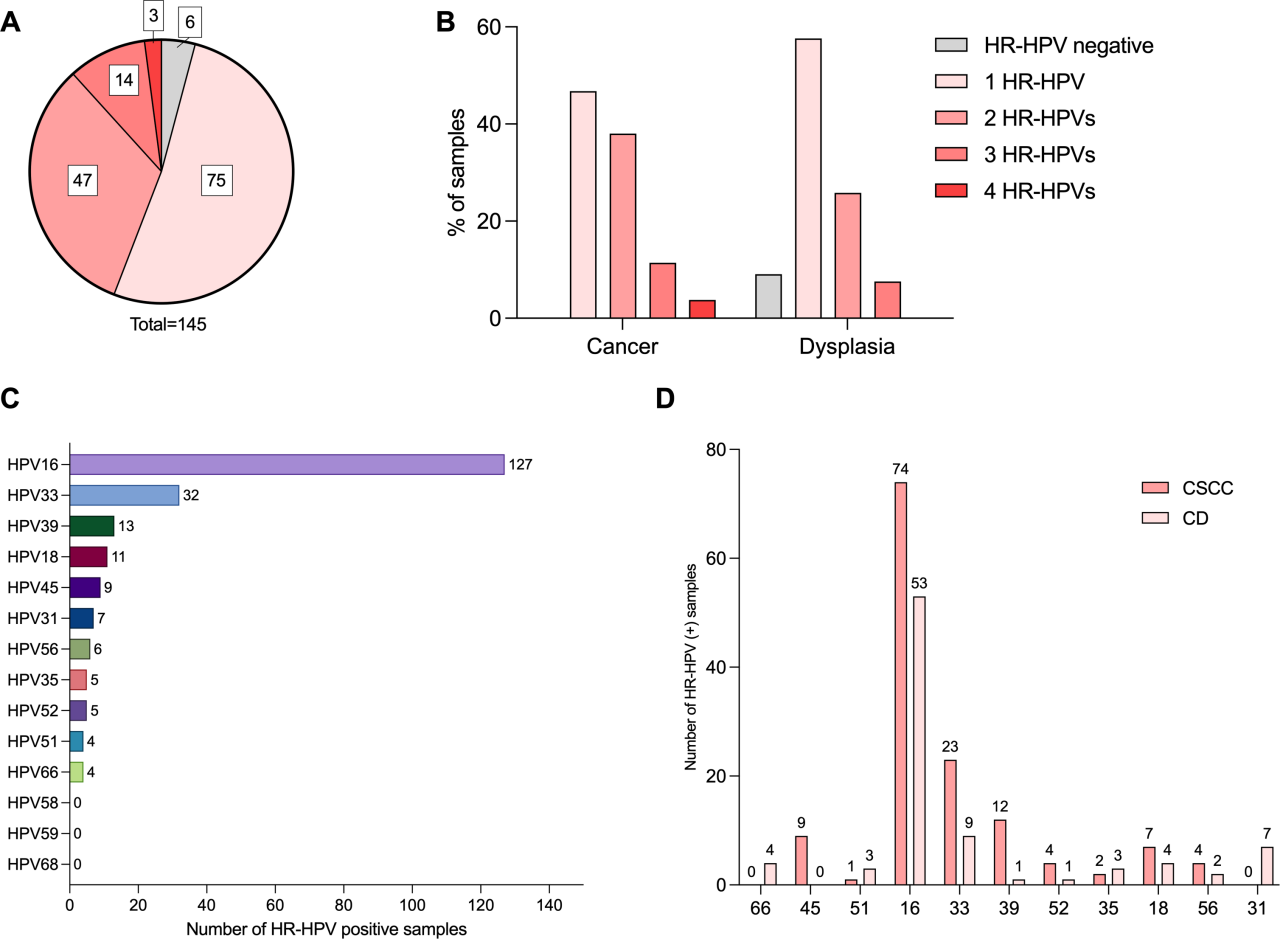
HR-HPV prevalence and genotyping data. **(A)** Number of HR-HPV in tissue sample overall; **(B)** by diagnosis [legend in panel **(B)** corresponds to panel **(A, B)**]. **(C)** HR-HPV genotype prevalence (number of positive samples) overall; **(D)** by diagnosis. HR-HPV, high-risk human papillomavirus; CSCC, cervical squamous cell carcinoma; CD, cervical dysplasia.

While most samples harbored one HR-HPV genotype (75/139, 53.9%), nearly half harbored multiple – 47/139 (33.8%) were positive for two HR-HPV genotypes, 14/139 (10.1%) for three, and 3/139 (2.2%) for four genotypes (**Figure 1A**). Most CSCC tissues harbored one (37/79, 46.8%) or two genotypes (30/79, 37.9%), but samples harboring the most HR-HPV genotypes, four, were exclusively from CSCC tissues (3/79, 3.8%). In CD samples, 63.3% were positive for one HR-HPV genotype (38/60), 28.3% were positive for two (17/60), and only 8.3% (5/60) for three (**Figure 1B**).

Notably, statistically significant differences were observed in the number of HR-HPV genotypes detected (one vs. multiple) in patients with CSCC and CD (p=0.0280). The cancerous samples were 2.19 times more likely to be positive for multiple HR-HPVs. When looking at CD and CSCC as a continuum of disease, disease severity (CIN I ➔ CIN II ➔ CIN III ➔ Grade 1 ➔ Grade 2 ➔ Grade 3) positively correlated with the number of HR-HPV detected (p=0.015, r=0.202, Spearman test).

#### 3.3.2 HR-HPV genotype prevalence in cervical cancer and dysplasia

The most prevalent genotype was HPV16 (127/139, 91.4%), followed by HPV33 (32/139, 23.0%), HPV39 (13/139, 9.4%), and HPV18 (11/139, 7.9%). All other genotypes were detected in fewer than 10 samples (**Figure 1C**). Three HR-HPV genotypes, HPV58, 59, and 68, were not detected.

HPV16 and HPV33 were the two most frequently detected genotypes, irrespective of the diagnosis. Prevalence of HPV16 was 93.6% (74/79) in CSCC and 88.3% (53/60) in CD cases, and of HPV33, 29.1% (23/79) in CSCC and 15% (9/60) in CD cases. The percentage of HPV16+ samples increased with the severity of both CD and CSCC. In CD samples, the percentage of HPV16+ samples increased from 67% (12/18) in CIN I to 86% (24/28) in CIN III, and in CSCC, from 71% (5/7) in grade 1 to 100% (26/26) in grade 3. HPV45 was detected in only in CSCC, and HPV66 and HPV31 only in CD cases (**Figure 1D**).

Considering that nearly half of the samples harbored multiple HR-HPV genotypes (64/139, 46.0%), we analyzed the most common combinations of the genotypes. Over half of the HPV16-positive samples were positive for HPV16 exclusively (68/127, 53.5%), while other genotypes were detected mainly in combination. Monoinfection with HPV45, 33, 52, 18, or 56 occurred in one sample each, and HPV35, in two samples. The most common HR-HPV combination overall was HPV16+HPV33+ (15/139, 10.8%) followed by HPV16+HPV18+ (8/139, 5.8%). The same was true for CSCC cases. In CD, the third most common combination was HPV16+HPV31+ (3/60, 5%).

#### 3.3.3 HR-HPV species in cervical cancer and dysplasia

Further, we decided to investigate a broader taxonomic unit, HPV species. The 14 HR-HPV genotypes investigated in this study belong to four HPV species – α5 (HPV51), α6 (HPV66, 56), α7 (HPV18, 45, 59, 39, 68), and α9 (HPV16, 31, 35, 33, 58, 52).

Most samples harbored HR-HPV genotypes belonging to only one species (100/139, 71.9%), followed by two (35/139, 25.2%) and three species (4/139, 2.9%). The majority were positive for α9 species only (97/139, 69.8%), or a α9+α7 combination (27/139, 19.4%). Other species combinations were present in fewer than ten samples. Interestingly, only three samples were α9 species negative, of which two were positive for α7, and one for α6 species.

As with the number of HR-HPVs per sample, CSCC tissues were more likely to harbor multiple HPV species (p=0.036). Additionally, we observed that the presence of genotypes belonging to both α9 and α7 species was more common in CSCC tissues, and the number of α9+α7 positive samples increased with higher disease severity. Overall, the number of species detected in the cervical tissue correlated with disease severity (p = 0.0005; r = 0.285).

#### 3.3.4 HR-HPV relative amount in cervical cancer and dysplasia

Combined use of AnyPlex and AllPlex kits allowed to semiquantify the amount of HR-HPV genotypes, based on low-medium-high approach used in AnyPlex. First, we looked at the overall HR HPV amount in the sample. If the sample was positive for multiple genotypes, we took the highest relative amount of HR HPV detected. Using this approach, we observed that more CSCC samples harbored high HR-HPV loads compared to CD samples – 51/79 (64.6%) vs. 27/60 (45.0%) (p = 0.025). Furthermore, the proportion of samples with high HR-HPV loads increased with disease severity. This was especially prominent in CD samples (CIN I: 2/14, 14.3%; CIN II: 8/20, 40%; CIN III: 17/26, 65.4%), with CIN III tissues more likely to harbor high HR HPV loads than CIN I (p=0.003).

Since HPV16 was the most common (127/139, 91.4%), we examined the HPV16 load more closely. More CSCC tissue samples harbored high HPV16 load, and were significantly more likely to harbor high HPV16 loads than CD samples (p=0.012). Additionally, the increase in samples harboring high HPV16 loads was again very prominent with increase in CD severity – from 1/12 (8.3%) in CIN I to 3/17 (17.6%) in CIN II, and to 15/24 (62.5%) in CIN III, where CIN III tissues were more likely to harbor high loads than CIN I (p=0.003).

Further, we revealed that overall HR-HPV load and HPV16 load significantly correlated with disease severity (p<0.001, r=0.288 and p<0.001, r=0.354, respectively; Spearman test). Additionally, positive correlation with disease severity was also observed for HPV39 (p<0.001, r=0.307), whilst for HPV66 and HPV31, the correlation was negative (p=0.027, r=-0.184; and p<0.001, r=-0.276, respectively).

### 3.4 HR-HPV genotype prevalence changes

Access to cervical tissue samples dating back to 2016, enabled us to monitor changes in HR-HPV genotype prevalence in CSCC and CD. We grouped the samples into three time periods: 2016-2018 (n=46, CSCC = 27/CD=19), 2019-2021 (n=54, CSCC = 26/CD=28), and 2022-2024 (n=45, CSCC = 26/CD=19) (**Table 1**).

**Figure 2:**
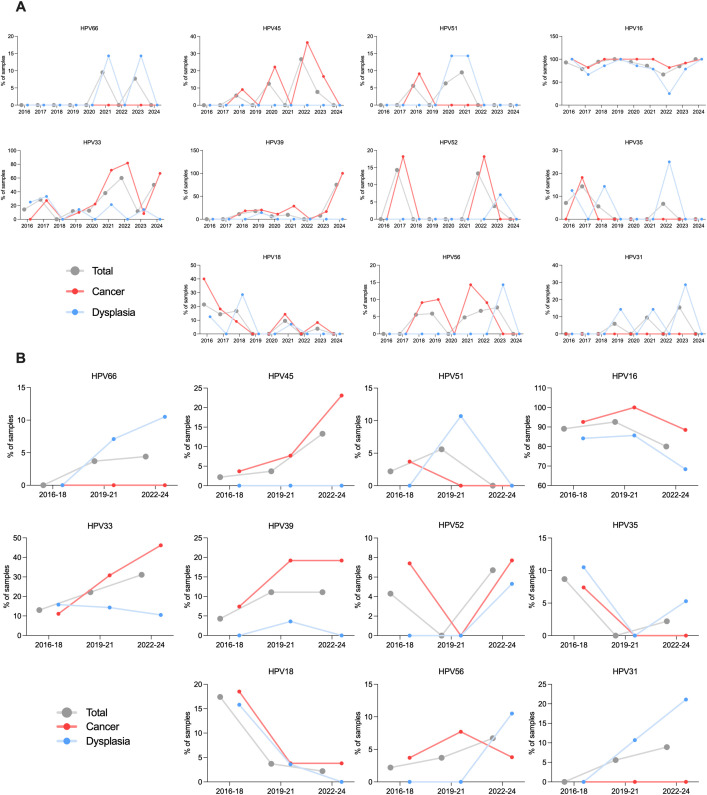
HR-HPV prevalence changes as a percentage of HR-HPV positive samples. **(A)** across the sampling years overall and by diagnosis; **(B)** across the three periods 2016-2018, 2019-2021, 2022–2024 overall and by diagnosis.

Overall, HPV16 prevalence decreased across the analyzed period. Before 2022, HPV16 was found in 89.1% to 92.6% of all samples (89.1% for 2016–2018 and 92.6% for 2019-2021). In 2022-2024, the prevalence of HPV16 decreased to 80.0%. A decrease in HPV16 prevalence was observed in both CSCC (2016-18: 92.6%; 2019-21: 100%; 2022-24: 88.5%) and CD cases (2016-18: 84.2%; 2019-21: 85.7%; 2022-24: 68.4%). An observable decrease in prevalence was also observed for HPV18 - from 17.4% in 2016–2018 to 2.2% in 2022-2024. According to Fisher’s exact test, samples from 2022 to 2024 were significantly less likely to be HPV18 positive than samples from 2016 to 2018 (p=0.032) (**Figure 2**).

HPV66, 45, 33, 39, and 31 appeared to increase in prevalence overall. In case of HPV45, 2.2% of the samples were positive for HPV45 in 2016-2018, and 13.3% in 2022-2024. Of note, HPV45 was detected only in CSCC cases. For HPV33, the prevalence increased from 13.0% in 2016–2018 to 31.1% in 2022-2024. The samples from 2022–2024 were three times more likely to be HPV33-positive than samples from 2016-2018 (Fisher’s exact test,p = 0.045). HPV39 sample positivity increased from 4.3% in 2016–2018 to 11.1% in both 2019–2021 and 2022-2024. HPV31 sample positivity increased from 0% in 2016–2018 to 8.9% in 2022-2024. In case of HPV66, the prevalence increased from 0% in 2016–2018 to 4.4% in 2022-2024 (**Figure 2**). Of note, both HPV31 and HPV66 were detected exclusively in CD cases.

## 4 Discussion

HR-HPV prevalence data is an important metric that provides insights not only into the epidemiological situation in the population, informing health policies, but also links to the disease severity, shaping treatment. In Latvia, HR-HPV prevalence data have mainly been collected by analyzing cervical smear samples from women undergoing routine cervical cancer screening. However, one of the first studies exploring HR-HPV genotype prevalence and association with cancer risk analyzed samples from women suffering from cervical cancer (between 1998 and 2001). They showed that the most common genotype in cancer was HPV16 (60.6%), followed by HPV18 (9%), HPV31 (4.5%), HPV45 (3.2%), and HPV33 (2.7%) (22). Our findings in CSCC samples are somewhat in line with these, but we have observed overall higher HR HPV prevalence rates. At the same time, HPV18 did not rank as the second most common genotype. Instead, the most common HR-HPV genotypes were HPV16 (94%), HPV33 (29%), HPV39 (15%), HPV45 (11%), and only then followed by HPV18 (11%). Our higher prevalence rates could be partly explained by the fact that we performed the analysis of DNA extracted from the cancer tissues directly, whereas the older study used cervicovaginal smears. This makes the former method more sensitive. Indeed, we have observed several CD cases which were negative for HR HPV in smears, but positive after analysis of DNA extracted from biopsies or conization materials (Isaguliants M, Spridzane A, unpublished observations). Overall, our data point at an increases in the prevalence of HPV33, 45, and 39, and decrease in the prevalence of HPV18.

Early studies have shown that HPV 31/33/45/52/58 positivity is associated with a significantly lower cumulative rate of CIN 3 or worse outcomes compared to HPV 16/18 (23). In 2015, Smelov V. et al. proposed a subdivision of genotypes into different risk groups: the highest risk oncogenic HPV types 16/18/31/33 with a CIN 3 or worse risk of 31.7%, the medium risk oncogenic HPV types, 35/45/52/58, with a CIN 3 or worse risk between 14% and 18%, and a large group (HPV 39/51/56/59/66/68) with limited risks, in which less than 10% of women developed CIN 3 or worse during the follow up period of the study (24). Later studies attributed the highest risk to HPV16, next tier after included HPV 31, 18, 33, 58, 52, and 45, while the lesser-risk tier included HPV 39, 51, 56, 59, and 68 as well as 66 (25). From 2021 WHO guidelines recommend distinguishing HPV16 and 18 from other high-risk genotypes, a concept dubbed extended genotyping (xGT) (26). According to xGT, high to moderate risk (depending on clustering) is attributed to HPV31/33/45/58, and lower risk to HPV 39/66 infections, all listed HR-HPVs among the ones for which we have recorded an increase in prevalence in CD or CC, or both. Jointly, prevalence of HR-HPVs from the moderate risk tier has increased from 2016–2018 to 2022–2024 by 37% and of HR-HPVs associated with low risk, by 7%. With this, our data fall in the general trend observed for the global HR HPV prevalence, i.e. a decrease of HPV16/18 (the first included into HPV vaccine) and increase in the prevalence of other HR-HPVs, specifically of moderate risk.

It is interesting to compare our results collected in women with cervical disease, with results of the prevalence studies, collected in conditionally healthy women, with respect to the prevalence of individual HR-HPVs. The most recent article on HR-HPV prevalence in Latvia among women undergoing routine screening (between 2022 and 2024) also reported HPV16 as the most common genotype (19.2%), however, in prevalence it was followed by HPV68 (13.4%), HPV31 (11.6%), and HPV66 (10%) (27), corroborating our data on increased prevalence, but only with regards to HPV31. Just like the 2025 study (27), a study exploring HR-HPV prevalence in the four Nordic countries in high-grade lesions and cervical cancer, also found HPV31 as one of the most common genotypes (28). Of note, we detected HPV31 in 5% of samples, and only in CD samples, which in part could be explained by the age of our study population, since HPV31 is more prevalent in younger women (29). The observed prevalence of HPV31 (and other non-HPV16/18 genotypes) could have been impacted by the age of our cohort. Studies have demonstrated that the proportion of HPV16/18 positive patients suffering from cancer and high-grade lesions decreases with age, while positivity for other genotypes increases (30, 31), highlighting the need for further studies including broader age groups.

A discrepancy in the prevalence was observed between our study and other prevalence studies with regard to HPV66 and HPV68. In our study, only 2.9% were HPV66-positive, and none were HPV68-positive. Interestingly, our HPV66-positive samples were exclusively from CD patients. Other recent studies also primarily associate HPV66 with normal cytology and low-grade lesions (32, 33). Since the timeline between HPV infection and the development of abnormal cervical cytology ranges from months to multiple years (34–38), our results and those of the 2025 study (27) may suggest that HPV 66 and 68 could be emerging contributors to CD and CC in the future. Further research on CD and CSCC tissues is necessary to characterize the prevalence of these two genotypes in dynamics and pinpoint their role in cervical disease.

In recent years, HPV33 has been reported as one of the most common genotypes, consistent with our findings in the retrospective cohort. Interestingly, not only has the prevalence been reported to be higher, but several studies have also demonstrated that HPV33 may also be associated with a higher risk of cervical disease development. In a study from 2020, HPV33 was associated with the second-highest risk of disease progression after HPV16. Namely, it has a cumulative risk of 18.4% progression to CIN III+ by year seven (39). Another study demonstrated that HPV33-positive patients had a higher incidence of CIN II+ lesions, and HPV33-positivity was a significant predictor of the probability of developing CIN II+ (40). While we found only one HPV33 exclusively positive sample, the continued decrease of HPV16 and increase in HPV33 prevalence may contribute to the emergence of mono-HPV33-positive patients with an increased risk of progression of cervical disease instead of clearance.

It is also interesting to note the complete absence among Latvian women with cervical disease of HPV58. HPV58 is high in the East Asia, particularly in China, South Korea, and Japan, where it is a significant contributor to cervical cancer cases and lesions, ranking as the third most common HR-HPV genotype in the region. This differs significantly from its lower prevalence globally, where it accounts for a much smaller percentage of cervical cancers (41, 42). The high prevalence in the East Asia and absence from Europe is thought to be linked to factors such as host genetics and local circulation of specific HPV58 variants (43, 44).

In this study, we found a high proportion of samples harboring not only multiple HR-HPV genotypes, but also multiple HPV species. This was especially prominent in CSCC samples, where 53.2% of samples were positive for multiple genotypes: CSCC samples were more likely to harbor multiple HR-HPVs, and the number of genotypes positively correlated with disease severity. Furthermore, 35.4% of CSCC samples harbored genotypes belonging to multiple species. Previous studies have demonstrated similar trends, namely, an increased risk of progression from low- to high-grade cervical lesions and cancer, and decreased patient survival for carriers of multiple HR HPVs species (45–51). Additionally, we demonstrated that CSCC samples were more likely to simultaneously harbor specific HR-HPV species, namely, α9 and α7, the prevalence of such co-infections increasing with increase in disease severity. Our data are in line with a study by Kaliff M et al. who had shown that simultaneous presence of HR HPVs belonging to both α9 and α7 increases the rate of cancer recurrence (52).

While some studies call for more time to observe significant HR-HPV genotype changes (10), other report changes (decreases and increases) in a number of HR-HPV genotypes early after the introduction of HPV vaccination (7–9). We have observed changes in the prevalence for several of the HR-HPVs, namely a significant decrease in the prevalence of HPV18, and a tendency to decrease for HPV16. However, several factors prevented us from attributing the observed changes to the direct effects of vaccination. Firstly, we had no access to vaccination records of the patients. Based on the age of participants, only two were in the age group eligible for national vaccination when it was started in 2010; thus, the majority were most likely unvaccinated. Although HPV vaccine is commercially available to anyone and more study participants than one could have been vaccinated, the probability of such an event is low, since repeated screenings of the attitude to HPV vaccination in Latvia have shown low acceptance and confidence in the HPV vaccine (53–55). Secondly, the process of cervical cancer development can take several decades, development of CINI from primary infection in 1–2 years, and to CINII in 3 to 5 years (56). With this, our results reflect an infection which have occurred 5 to 10 years before sampling, for the earliest samples, at the time of on-start of HPV vaccination in 2010, and for the latest, 10 years later. Considering that most of the women in the cohort were not vaccinated, we have associated the observed changes in HR HPV prevalence, specifically the significant reduction in the prevalence of HPV18 and a reduction for HPV16, with indirect effects of vaccination on the non-vaccinated population (herd effects). Overall, the changes we observed in the prevalence of HR HPVs in women with cervical disease highlight the absolute necessity of vaccination with multi-valent vaccines with reinforcement of the national HPV vaccination efforts beyond current vaccination coverage of 62% (57) to spare generations of women the suffering from preventable disease caused by HPV16 and non-HPV16/18 HR-HPVs.

High cervical cancer incidence and mortality rates accompanied by low screening and vaccination coverage present a serious problem for Latvia (3). Cervical cancer screening in Latvia was initiated in 2009 based on cervical cytology, but since mid-2022, screening has transitioned to primary HR-HPV screening (58). Our results have demonstrated the continued usefulness of HR-HPV genotyping and the benefits it can provide, not only for early diagnosis of cervical transformation, but also for understanding the epidemiology and disease association of HR-HPVs circulating in the population, for example, for patient stratification. We demonstrated that both the presence of multiple genotypes and species, as well as specific genotypes and their loads, such as HPV16 and 39, are associated with disease severity, with patients harboring these combinations potentially requesting personalized schemes of follow-up, and early treatment of the lesions. Furthermore, we have demonstrated that while HPV16 remains the most prevalent genotype, multiple others, such as HPV66, 45, 33, 39, and 31 have replaced HPV18. Their role in the induction of cervical disease across the world is becoming more and more prominent (59–61). This trend should be taken into account in shaping national HPV screening and HPV vaccination policies.

## 5 Study limitations

Even though prevalence data in Latvia, especially in women already suffering from cervical disease, is lacking, and our study helps to fill these gaps, we have to list a number of limitations of the data we have acquired.

The reported study was an uninterventional retrospective study, with access to information on patient age, diagnosis, and histological evaluation, but not any demographic information or other risk factors that could have influenced HR-HPV prevalence. Furthermore, the study population was small and limited to patients residing in the capital of Latvia, Riga, which may not represent the whole Latvian population. By today, Riga has 591,882 inhabitants, and Latvia, 1,853,559 people (62). With this, population-wise, the data collected in Riga represent data of >30% of the Latvian population; however, the habits and risk behaviors of the population in the capital differ from those of the smaller cities and of the rural population. Altogether, the lack of other demographic data, the limited sample size, and the geographical area of sample collection may have a negative impact on the statistical power of our analysis of changes in HR-HPV prevalence among women with cervical disease, and their attribution to Latvia in general. Further studies with bigger sample sizes from each year and different territories in the country as well as the potential inclusion of cervical cancers other than squamous cell carcinomas (such as cervical adenocarcinomasare necessary to make general conclusions on the changes in the prevalence of HR-HPV genotypes in Latvia.

Very few of the study participants could have received HPV vaccine (estimated based on their age, as no vaccination data was available from the patient registry). Due to this, our data do not allow to draw conclusions on the direct effects of HPV vaccination on HR-HPV prevalence in Latvia. Still, we detected a statistically significant decrease in the prevalence of HPV18, and a decrease in the prevalence of HPV16, although it did not reach the level of significance, which we have tentatively explained by the herd effect.

Another limitation raising from the limited amount of samples analyzed, concerns the combinations of detected HR-HPV genotypes. Even though we were able to detect a variety of HR-HPV combinations, each of them represented a very small part of the cohort, thus we were unable to evaluate association of specific HR-HPV genotype combinations with disease severity.

While analyzing associations between HR-HPV genotypes and their load and the severity of cervical disease, we used the results of two HR-HPV DNA tests, semi-quantitative AnyPlex and quantitative AllPlex. Throughout data analysis, the results of the quantitative test were translated into semi-quantitative results for overall cohort analysis using a calibration curve built with HPV16 DNA (19). No curves were built for other HR-HPV genotypes. With this, translation of the data from quantitative into semiquantitative test could have been not 100% accurate. Furthermore, for the samples analyzed by both AnyPlex and AllPlex tests, the AnyPlex test demonstrated lower viral loads. This “technical” reduction of the virus load in part of the samples assessed by AnyPlex and/or translated to AnyPlex, specifically in samples with comparatively low viral load, could have reduced the significance of associations between viral load and severity of cervical disease. This, however, indicates that the ones we have observed, despite this discrepancy, were of high significance, i.e., the difference between the performance of two tests did not affect study results.

## Data Availability

The raw data supporting the conclusions of this article will be made available by the authors, without undue reservation.
